# Association Between Cumulative Sedative Exposure and ICU Length of Stay Without a Significant Association with Mortality

**DOI:** 10.3390/life16050833

**Published:** 2026-05-19

**Authors:** Josef Yayan

**Affiliations:** Department of Internal Medicine, Division of Pulmonary, Allergy, and Sleep Medicine, Helios University Hospital Wuppertal, Witten/Herdecke University, Heusnerstr. 40, 42283 Wuppertal, Germany; josef.yayan@hotmail.com; Tel.: +49-020-2896-3936; Fax: +49-020-2896-3901

**Keywords:** sedation, intensive care, ICU length of stay, critical care, clinical outcomes, sedative exposure, sedatives, critical illness

## Abstract

Background: Sedative medications are widely used in intensive care units (ICUs) to facilitate patient management; however, their association with clinical outcomes remains incompletely understood. This study aimed to evaluate the association of cumulative sedative exposure with ICU length of stay (LOS) in a large cohort of critically ill adult patients. Methods: A retrospective observational study was conducted using the Medical Information Mart for Intensive Care IV (MIMIC-IV) database. Adult ICU patients (≥18 years) with documented sedative administration were included. Total sedative exposure was quantified as the cumulative dose administered during the ICU stay. The primary outcome was ICU LOS, while the secondary outcome was in-hospital mortality. Patients were stratified into quartiles according to cumulative sedative dose. Multivariable regression analysis was performed to assess the association between total sedative exposure and ICU LOS. Results: A total of 2953 ICU stays were analyzed. Higher cumulative sedative exposure was associated with significantly prolonged ICU LOS. Mean ICU LOS increased from 68.75 h in the lowest quartile to 250.65 h in the highest quartile (*p* < 0.001). A weak positive correlation was observed between log-transformed total sedative dose and ICU LOS (r = 0.33, *p* < 0.001). In multivariable analysis, cumulative sedative exposure remained significantly associated with ICU LOS (β = 15.79, 95% CI 14.17–17.41, *p* < 0.001). No consistent association was identified between sedative exposure and in-hospital mortality. Conclusions: Higher cumulative sedative exposure was associated with longer ICU LOS but not with increased in-hospital mortality. These findings support the importance of carefully tailored sedation strategies in critically ill patients. However, due to the retrospective observational design, causality cannot be inferred, and residual confounding related to illness severity and treatment duration may remain.

## 1. Introduction

Sedative medications are widely used in intensive care units (ICUs) to facilitate mechanical ventilation, reduce patient discomfort, and improve tolerance to invasive procedures [1]. Commonly administered sedative agents include propofol, benzodiazepines, and dexmedetomidine, each characterized by distinct pharmacodynamic properties and safety profiles [2].

Despite their important clinical benefits, sedative medications have been associated with adverse outcomes, including prolonged mechanical ventilation, delirium, and prolonged ICU length of stay (LOS) [3,4]. In particular, excessive or prolonged sedation may contribute to delayed recovery, prolonged ICU dependency, and increased healthcare resource utilization [5]. Consequently, current critical care guidelines recommend minimizing unnecessary sedative exposure and favoring lighter sedation strategies whenever clinically feasible [6].

Nevertheless, real-world sedation practices remain highly variable across ICU settings, and the relationship between cumulative sedative exposure and clinical outcomes remains incompletely understood. Previous studies have primarily focused on sedation depth, daily sedation interruption, or protocol-based sedation strategies, whereas relatively fewer investigations have quantitatively evaluated the association between total cumulative sedative dose and ICU outcomes in large real-world cohorts [7].

Importantly, sedation requirements are strongly influenced by illness severity, mechanical ventilation status, underlying comorbidities, and ICU treatment strategies, all of which may additionally affect patient outcomes and ICU LOS [8]. Therefore, observational analyses examining sedative exposure are potentially susceptible to residual confounding and reverse causation, in which prolonged ICU stays may themselves result in higher cumulative sedative exposure. Large critical care databases such as the Medical Information Mart for Intensive Care IV (MIMIC-IV) database provide an opportunity to investigate these associations in diverse ICU populations using real-world clinical data [9].

Therefore, this study aimed to evaluate the association between cumulative sedative exposure and ICU LOS in a large cohort of critically ill adult patients. In addition, the relationship between cumulative sedative exposure and in-hospital mortality was examined. Higher cumulative sedative exposure was hypothesized to be associated with prolonged ICU LOS, but not necessarily with higher in-hospital mortality.

## 2. Materials and Methods

### 2.1. Data Source and Study Population

This retrospective observational study was conducted using data from the Medical Information Mart for Intensive Care IV (MIMIC-IV, version 3.1) database (Massachusetts Institute of Technology, Cambridge, MA, USA), a large publicly available single-center care database containing de-identified clinical data from patients admitted to intensive care units at the Beth Israel Deaconess Medical Center (Boston, MA, USA) between 2008 and 2019. The database includes detailed information on patient demographics, diagnoses, procedures, medication administration, laboratory findings, and ICU stay characteristics. Data extraction and processing were performed using Google BigQuery (Google Cloud Platform; Google LLC, Mountain View, CA, USA), which enabled structured querying and aggregation of relevant variables.

Access to the MIMIC-IV database was obtained after completion of the required data use agreement and credentialing process in accordance with database regulations. Because all patient data were fully de-identified, informed consent was waived.

Adult patients (≥18 years) with at least one ICU admission and documented administration of sedative medications were included in the analysis. ICU stays with missing data for key variables were excluded to improve data completeness and analytical reliability. Each ICU stay was analyzed independently to account for variability across repeated ICU admissions.

### 2.2. Data Extraction and Variable Definition

Data extraction was performed using structured query language (SQL) within the BigQuery environment. Relevant tables included patient demographic data, ICU stay characteristics, and medication administration records.

Sedative exposure was defined as the total cumulative sedative dose administered during the ICU stay. Commonly used sedative agents included propofol, benzodiazepines, and dexmedetomidine. Due to differences in pharmacological properties and dosing regimens among sedative classes, cumulative exposure was analyzed as an aggregate measure of overall sedative administration. The potential limitations associated with combining different sedative agents were considered when interpreting the results. Because the distribution of cumulative sedative dose was highly skewed, logarithmic transformation was applied for subsequent statistical analyses.

The primary outcome was ICU length of stay (LOS), defined as the time interval between ICU admission and ICU discharge, expressed in hours (h). The secondary outcome was in-hospital mortality.

To evaluate potential dose–response relationships, patients were stratified into quartiles according to total cumulative sedative dose.

### 2.3. Statistical Analysis

Continuous variables are presented as mean ± standard deviation (SD) or median with interquartile range (IQR), as appropriate. Categorical variables are presented as percentages. Group comparisons were performed using the Mann–Whitney U test for non-normally distributed variables.

The association between cumulative sedative dose and ICU LOS was assessed using Pearson correlation analysis following logarithmic transformation of the sedative dose variable. Multivariable linear regression analysis was performed to evaluate the independent association between cumulative sedative exposure and ICU LOS. The regression model included log-transformed cumulative sedative dose and age as covariates.

Because of the retrospective observational design and limitations in the availability of complete clinical severity variables, residual confounding related to illness severity, mechanical ventilation duration, comorbidities, and ICU treatment characteristics cannot be excluded. Therefore, the findings should be interpreted as associative rather than causal relationships.

Results are reported as regression coefficients (β) with 95% confidence intervals (CI). All statistical analyses were conducted using R statistical software (R Foundation for Statistical Computing, Vienna, Austria), version 4.3.1. A two-sided *p*-value < 0.05 was considered statistically significant.

## 3. Results

### 3.1. Study Population

A total of 2953 ICU stays were included in the final analysis following data cleaning and aggregation procedures. Baseline characteristics of the study population are summarized in Table 1.

The mean age of the cohort was 36.6 ± 23.8 years, with a median age of 28.0 years (IQR 23.0–31.0), indicating a relatively young ICU population compared with many general critical care cohorts. This age distribution may reflect a higher proportion of trauma-related or surgical ICU admissions within the analyzed dataset. Male patients accounted for 57.1% of the cohort, whereas female patients represented 42.9%.

The median total cumulative sedative dose administered during the ICU stay was 1215.8 (IQR 24.2–5022.2), indicating substantial variability in sedative exposure among patients. Mean ICU length of stay (LOS) was 119.6 ± 170.0 h, indicating in clinical courses and ICU resource utilization. The overall in-hospital mortality rate was 12.0% (Table 1).

### 3.2. Association Between Total Sedative Dose and ICU Length of Stay

Patients were stratified into quartiles according to total cumulative sedative dose to evaluate potential dose–response relationships. An association between increasing sedative exposure and prolonged ICU LOS was observed.

Mean ICU LOS demonstrated a progressive increase across sedative dose quartiles, rising from 68.75 h in the lowest quartile (Q1) to 250.65 h in the highest quartile (Q4), corresponding to an approximately fourfold increase in ICU stay duration (Table 2). Intermediate quartiles showed a gradual increase in ICU LOS, supporting a suggested dose-dependent association between cumulative sedative exposure and prolonged ICU LOS.

Because of the observational study design, these findings should be interpreted as associative rather than causal. In particular, patients with longer ICU stays or more severe illness may have required prolonged sedation, potentially contributing to reverse causation.

The difference in ICU LOS between the lowest and highest quartiles was statistically significant (*p* < 0.001), indicating that patients receiving higher cumulative sedative doses tended to experience prolonged ICU LOS.

**Figure 1:**
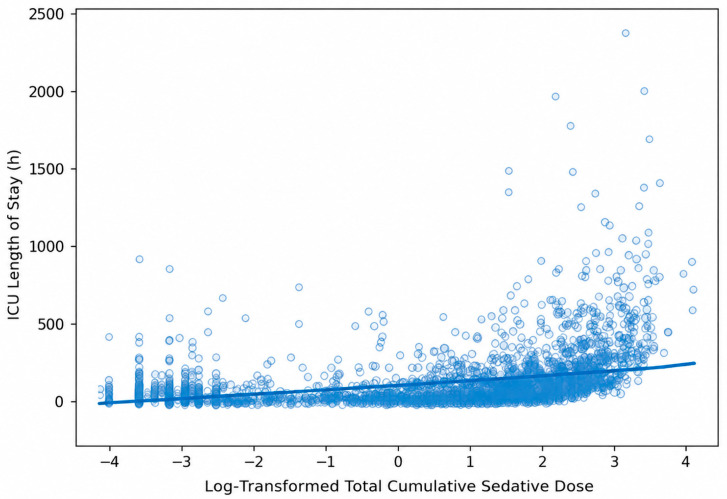
Association between log-transformed total cumulative sedative dose and ICU length of stay (LOS). Weak positive correlation was observed between log-transformed total cumulative sedative dose and ICU LOS (r = 0.33, *p* < 0.001).

**Figure 2:**
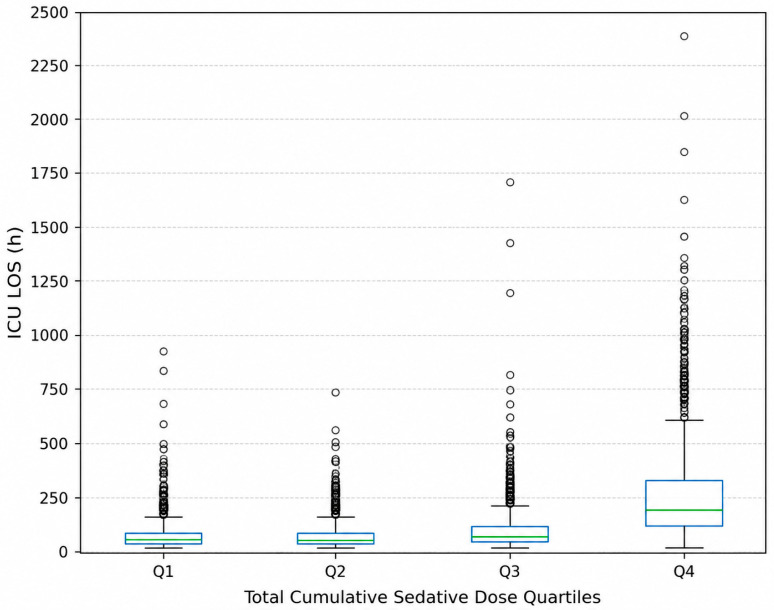
ICU length of stay (LOS) stratified by total cumulative sedative dose quartiles. An overall increase in ICU LOS was observed across sedative dose quartiles, with highest exposure group (Q4) demonstrating markedly prolonged ICU stays compared with the lowest quartile (Q1) (*p* < 0.001).

This association was further supported by correlation analysis, which showed a weak positive relationship between log-transformed total cumulative sedative dose and ICU LOS (r = 0.33, *p* < 0.001) (Figure 1). Boxplot analysis additionally demonstrated a progressive increase in ICU LOS across quartiles, with the greatest variability and most extreme values observed in the highest exposure group (Q4) (Figure 2).

Because cumulative sedative exposure may increase with prolonged ICU treatment, the possibility of reverse causation should be considered when interpreting these findings.

### 3.3. Multivariate Analysis

To evaluate whether the observed association between cumulative sedative exposure and ICU LOS was independent of baseline demographic factors, a multivariable regression model was constructed.

After adjustment for age, log-transformed total cumulative sedative dose remained significantly associated with ICU LOS (β = 15.79, 95% CI 14.17–17.41, *p* < 0.001) (Table 3). Specifically, each unit increase in log-transformed cumulative sedative dose was associated with an approximate 15.8 h increase in ICU LOS.

In contrast, age was not significantly associated with ICU LOS in the adjusted model (β = 0.18, 95% CI −0.06 to 0.43, *p* = 0.14) (Table 3).

Because the regression model included only limited covariates, residual confounding related to illness severity, duration of mechanical ventilation, comorbidities, ICU subtype, and indication for sedation cannot be excluded. Therefore, the observed association between cumulative sedative exposure and ICU LOS should not be interpreted as evidence of causal relationship.

### 3.4. Distribution of Total Sedative Exposure

**Figure 3:**
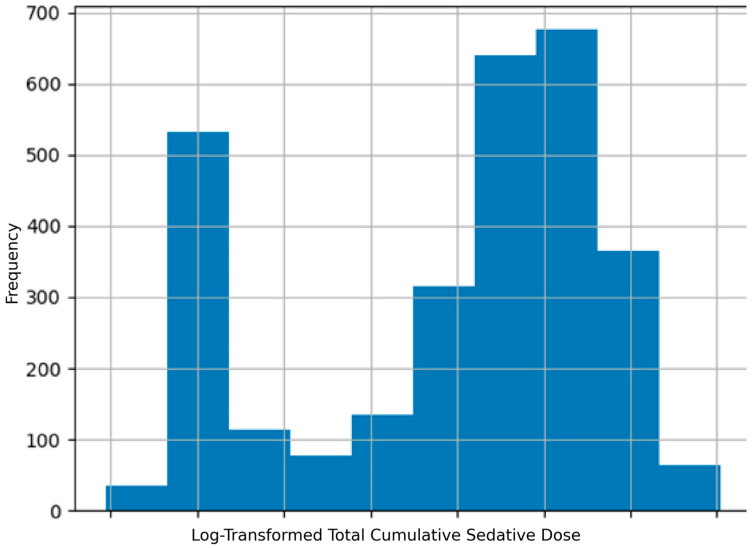
Distribution of log-transformed total cumulative sedative dose among ICU patients demonstrating a right-skewed exposure pattern.

The distribution of total cumulative sedative dose was highly skewed, with a pronounced right-skewed tail indicating that a subset of patients received substantially higher sedative doses. Owing to this non-normal distribution, logarithmic transformation was applied for subsequent correlation and regression analyses (Figure 3).

The broad variability in cumulative sedative exposure likely reflects heterogeneity in clinical practice across ICUs, underlying illness severity, requirements for mechanical ventilation, sedation strategies, and individual patient characteristics. These findings additionally emphasize the complexity of interpreting cumulative sedative exposure in observational critical care cohorts.

### 3.5. In-Hospital Mortality

In-hospital mortality rates varied across quartiles of total cumulative sedative dose, ranging from 9.0% to 16.0%, without demonstrating a clear dose–response relationship (Table 2). Although the second quartile (Q2) showed the highest mortality rate, the highest exposure group (Q4) did not show a proportional increase in mortality.

These findings suggest that cumulative sedative exposure was more strongly associated with prolonged ICU LOS than with in-hospital mortality in this cohort. However, mortality outcomes in critically ill patients are influenced by multiple clinical factors, including illness severity, comorbidities, organ dysfunction, and underlying indications for sedative administration. Therefore, the absence of a clear association between cumulative sedative exposure and mortality should be interpreted cautiously.

## 4. Discussion

In this large retrospective cohort study, higher cumulative sedative exposure was significantly associated with prolonged ICU length of stay (LOS). Patients in the highest quartile of total cumulative sedative dose experienced nearly a fourfold increase in ICU LOS compared with patients in the lowest quartile. This association remained statistically significant after adjustment for age in multivariable analysis, suggesting that greater sedative exposure was associated with prolonged ICU stay in this cohort.

These findings are consistent with previous studies demonstrating that excessive or prolonged sedation may delay recovery and contribute to extended ICU stays [10,11]. Deep sedation has been associated with delayed liberation from mechanical ventilation, increased risk of delirium, prolonged cognitive dysfunction, and impaired neurological recovery, all of which may contribute to prolonged ICU LOS [12,13]. Current ICU sedation guidelines therefore recommend light sedation strategies whenever clinically appropriate and minimizing unnecessary sedative exposure [1,2,14]. Our findings extend previous observations by quantitatively examining cumulative sedative exposure in a large real-world ICU dataset.

The observed weak positive correlation between log-transformed cumulative sedative dose and ICU LOS further supports an association between greater sedative exposure and prolonged ICU stay. While previous investigations have mainly focused on sedation depth, daily sedation interruption, or protocol-based sedation strategies, relatively fewer studies have evaluated cumulative sedative exposure as a continuous quantitative variable [15]. The progressive increase in ICU LOS across sedative dose quartiles observed in the present study further supports this association.

However, several important considerations should be acknowledged when interpreting these findings. First, because of the retrospective observational design, causality cannot be established. Patients with more severe illness or prolonged requirements for mechanical ventilation are more likely to require higher sedative exposure, which may contribute to prolonged ICU LOS independently of sedation itself. Consequently, the possibility of reverse causation remains, whereby prolonged ICU treatment leads to increased cumulative sedative administration, rather than sedative exposure directly causing prolonged ICU LOS.

Second, although multivariable analysis adjusted for age, important potential confounders such as illness severity scores (e.g., SOFA or APACHE), mechanical ventilation duration, ICU subtype, comorbidities, and indication for sedation were not uniformly available for inclusion in the regression model. Therefore, residual confounding cannot be excluded.

Another important limitation relates to the aggregation of different sedative agents into a single cumulative exposure variable. Sedative agents, such as propofol, benzodiazepines, and dexmedetomidine, possess distinct pharmacologic profiles, recovery characteristics, and adverse effect patterns that may differentially influence patient outcomes. Consequently, the present analysis reflects overall sedative exposure rather than the effects of individual sedative classes.

Interestingly, despite the strong association between cumulative sedative exposure and ICU LOS, no consistent association with in-hospital mortality was observed. This finding is consistent with previous reports suggesting that sedation strategies may primarily affect intermediate ICU outcomes, including duration of mechanical ventilation and ICU LOS, rather than mortality itself [16]. Mortality in critically ill patients is multifactorial and strongly influenced by illness severity, organ dysfunction, underlying diagnoses, and comorbid conditions [17], potentially obscuring independent associations with sedative exposure.

The relatively young age profile of the present cohort should also be considered when interpreting the findings. Compared with many general ICU populations, the analyzed cohort demonstrated a lower median age, which may reflect a greater proportion of trauma-related or surgical ICU admissions within the MIMIC-IV dataset [18]. In addition, the single-center nature of the database may limit generalizability to other ICU settings and older critically ill populations.

From a clinical perspective, these findings support current guideline recommendations advocating careful titration and minimization of sedative exposure whenever feasible. Strategies including protocolized sedation management, daily sedation interruption, and light sedation targets have previously been associated with improved ICU outcomes and shorter ICU stays. Taken together, these findings highlight the potential clinical relevance.

## 5. Limitations

This study has several important limitations. First, the retrospective observational design does not allow causal inference, and residual confounding cannot be excluded despite multivariable adjustment. Unmeasured variables, including illness severity, organ dysfunction, comorbidities, mechanical ventilation duration, ICU subtype, and clinical decision-making, may have influenced both cumulative sedative exposure and ICU outcomes.

Second, the analysis was based on a single-center database, which may limit the generalizability of the findings to other institutions with different patient populations, sedation practices, and ICU management strategies.

Third, detailed information regarding illness severity scores, such as the Sequential Organ Failure Assessment (SOFA) score or Acute Physiology and Chronic Health Evaluation II (APACHE II) score, was not uniformly available for inclusion in the current analysis. Similarly, data regarding sedation indications, sedation depth, and standardized sedation targets were not comprehensively captured. These factors may substantially affect both sedative requirements and patient outcomes.

Fourth, the present study evaluated total cumulative sedative exposure without differentiating between specific sedative classes or drug combinations. Sedative agents such as propofol, benzodiazepines, and dexmedetomidine exhibit distinct pharmacologic properties, adverse effect profiles, and recovery characteristics that may differentially affect ICU outcomes.

Fifth, reverse causation remains an important consideration. Patients with prolonged ICU stays or greater illness severity may inherently require longer durations and higher cumulative sedative doses. Therefore, higher cumulative sedative exposure may partly reflect greater clinical complexity rather than a direct effect of sedative exposure itself.

Finally, the relatively young age distribution of the analyzed cohort may limit the applicability of the findings to older and more general ICU populations.

Future studies incorporating standardized illness severity scores such as SOFA or APACHE, as well as mechanical ventilation duration, are warranted to further clarify these associations.

Despite these limitations, the large sample size and use of a comprehensive real-world critical care database provide valuable insights into sedation practices and their associations with ICU outcomes in critically ill patients.

## 6. Conclusions

In this large retrospective cohort study, higher cumulative sedative exposure was associated with prolonged ICU length of stay. Patients receiving the highest cumulative sedative doses experienced substantially longer ICU stays compared with patients with lower sedative exposure, suggesting an overall dose-dependent association.

This association remained statistically significant after adjustment for age; however, because of the observational study design and the potential influence of residual confounding factors, including illness severity and duration of mechanical ventilation, causality cannot be established. Reverse causation also remains possible, as patients requiring prolonged ICU treatment may inherently receive greater cumulative sedative exposure.

In contrast, no consistent association between cumulative sedative exposure and in-hospital mortality was observed.

These findings highlight the clinical relevance of carefully tailored sedation strategies in critically ill patients. Approaches aimed at minimizing unnecessary sedative exposure, including protocolized sedation management and light sedation strategies, may contribute to reduced ICU LOS and improved resource utilization. Future prospective and multicenter studies are warranted to further clarify causal associations and to identify optimal sedation practices across diverse ICU populations.

## Figures and Tables

**Table 1:** Baseline characteristics of study population. ICU LOS is reported in hours (h).

Variable	Value
N	2953
Age, years (mean ± SD)	36.6 ± 23.8
Age, years (median, IQR)	28.0 (23.0–31.0)
Male sex, *n (*%)	1686 (57.1)
Female sex, *n* (%)	1267 (42.9)
Total cumulative sedative dose, median (IQR)	1215.8 (24.2–5022.2)
ICU LOS, h (mean ± SD)	119.6 ± 170.0
In-hospital mortality, *n* (%)	354 (12.0)

**Table 2:** Association between total cumulative sedative dose quartiles and clinical outcomes. Comparison between Q1 and Q4 was performed using a Mann–Whitney U test (*p* < 0.001).

Quartile of Total Cumulative Sedative Dose	Mean ICU LOS, h	In-Hospital Mortality, %
Q1	68.75	11.0
Q2	68.21	16.0
Q3	90.98	9.0
Q4	250.65	12.0

**Table 3:** Multivariable linear regression analysis evaluating the association between total cumulative sedative dose and ICU length of stay (LOS).

Variable	β	95% CI Lower	95% CI Upper	*p*-Value
Log-transformed total cumulative sedative dose	15.79	14.17	17.41	<0.001
Age	0.18	−0.06	0.43	0.14

## Data Availability

The data used in this study are publicly available from the Medical Information Mart for Intensive Care IV (MIMIC-IV) database via PhysioNet, subject to completion of the required training and data use agreements.
